# Ectropion uveae in a pediatric patient with refractory NF1-associated glaucoma: a 4-year case report

**DOI:** 10.3389/fmed.2026.1829841

**Published:** 2026-09-09

**Authors:** Yulei Geng, Weijia Li, Kuitang Shi, Yawen Li, Tianyu Zhang, Jiaming Lu, Shuai Wang, Xiaowei Yan, Guangxian Tang, Hengli Zhang

**Affiliations:** Department of Ophthalmology, Shijiazhuang People's Hospital, Hebei, Shi Jia Zhuang, China

**Keywords:** case report, ectropion uveae, glaucoma drainage device, neurofibromatosis type 1, refractory glaucoma

## Abstract

**Background:**

Glaucoma secondary to neurofibromatosis type 1 (NF1) is a vision-threatening complication characterized by variable phenotypic expression. The prognostic significance of specific anterior segment signs, particularly ectropion uveae (EU), remains unclear.

**Case presentation:**

We report a 7-year-old girl with NF1 who presented with unilateral refractory glaucoma and prominent EU in the affected eye. Despite maximal medical therapy and sequential surgical interventions, including microcatheter-assisted transluminal trabeculotomy, subsequent scar tissue excision with goniotomy, and two sessions of transscleral cyclophotocoagulation, intraocular pressure (IOP) remained uncontrolled. Implantation of a glaucoma drainage device (GDD) resulted in initial IOP reduction, with IOP maintained at 16/18 mmHg during the first 3 months. At 1 year post-GDD, IOP was 23 mmHg, and brinzolamide was subsequently added.

**Conclusion:**

This 4-year longitudinal case highlights an association between prominent ectropion uveae and a refractory surgical course in this patient. Traditional angle surgeries appeared to carry a high risk of failure, whereas GDD implantation provided IOP reduction from 42 to 23 mmHg at 1 year, although adjunctive medication was subsequently required. This observation raises the question of whether earlier GDD implantation might prove beneficial in similar patients. However, this hypothesis requires validation in a larger series.

## 1 Introduction

Neurofibromatosis type 1 (NF1) is an autosomal-dominant multisystem disorder characterized by heterogeneous clinical manifestations involving the skin, nervous system, and eyes (1–3). Although Lisch nodules and optic pathway gliomas are well-recognized ocular features, NF1-associated glaucoma is a rare but devastating complication, occurring in approximately 1%–2% of patients (4). In NF1-associated glaucoma, the pathogenesis is complex and multifactorial, potentially involving angle infiltration by neurofibromas, synechial closure, or primary angle dysgenesis (5, 6). The diagnosis of NF1 is based on the revised 2021 international consensus criteria, which require the presence of at least two of the following features: six or more café-au-lait macules (>5 mm in prepubertal individuals or >15 mm in postpubertal individuals); two or more neurofibromas of any type or one or more plexiform neurofibromas; axillary or inguinal freckling; optic pathway glioma; two or more Lisch nodules; characteristic osseous lesions; choroidal anomalies; or a first-degree relative with NF1. Alternatively, the presence of a pathogenic NF1 gene variant is sufficient for diagnosis (7).

Ectropion uveae (EU), characterized by the anterior migration of iris pigment epithelium onto the iris surface, represents an uncommon but characteristic anterior segment finding in NF1 (8, 9). Ectropion uveae is a rare condition in the general population. Among patients with NF1, its prevalence has been estimated at approximately 1.2% in a large cohort study (8). Ectropion uveae itself requires no specific treatment; its clinical significance lies in the associated glaucoma, which is managed according to standard antiglaucoma strategies, ranging from medical therapy to surgical intervention. It is often regarded as evidence of developmental anomaly (10). Histopathological studies suggest EU in NF1 may be associated with a proliferative endothelial membrane covering the angle and iris, contributing to mechanical outflow obstruction. However, the clinical significance of EU in relation to the severity of glaucoma and, more critically, to the failure of conventional surgical interventions, remains unclear.

This longitudinal case study details a 4-year management course of a pediatric NF1 patient with refractory glaucoma and prominent EU, culminating in intraocular pressure (IOP) reduction with a glaucoma drainage device (GDD). Compared with previous reports, our case provides a longer follow-up period documenting the stepwise failure of multiple angle surgeries prior to GDD implantation.

## 2 Case presentation

A 7-year-and-5-month-old girl was referred to our department in June 2021 for progressive corneal enlargement and buphthalmos of the left eye over a 2-month period. Her parents reported occasional left orbital pain and ipsilateral headache. Initial evaluation at a local hospital revealed an IOP >50 mmHg in the left eye, leading to a diagnosis of glaucoma managed with maximally tolerated medical therapy. The patient’s past ocular history was significant for left eye amblyopia, treated with atropine penalization for 5 years. She was born full-term via cesarean section without perinatal complications and denied any systemic illness or trauma.

**Figure 1:**
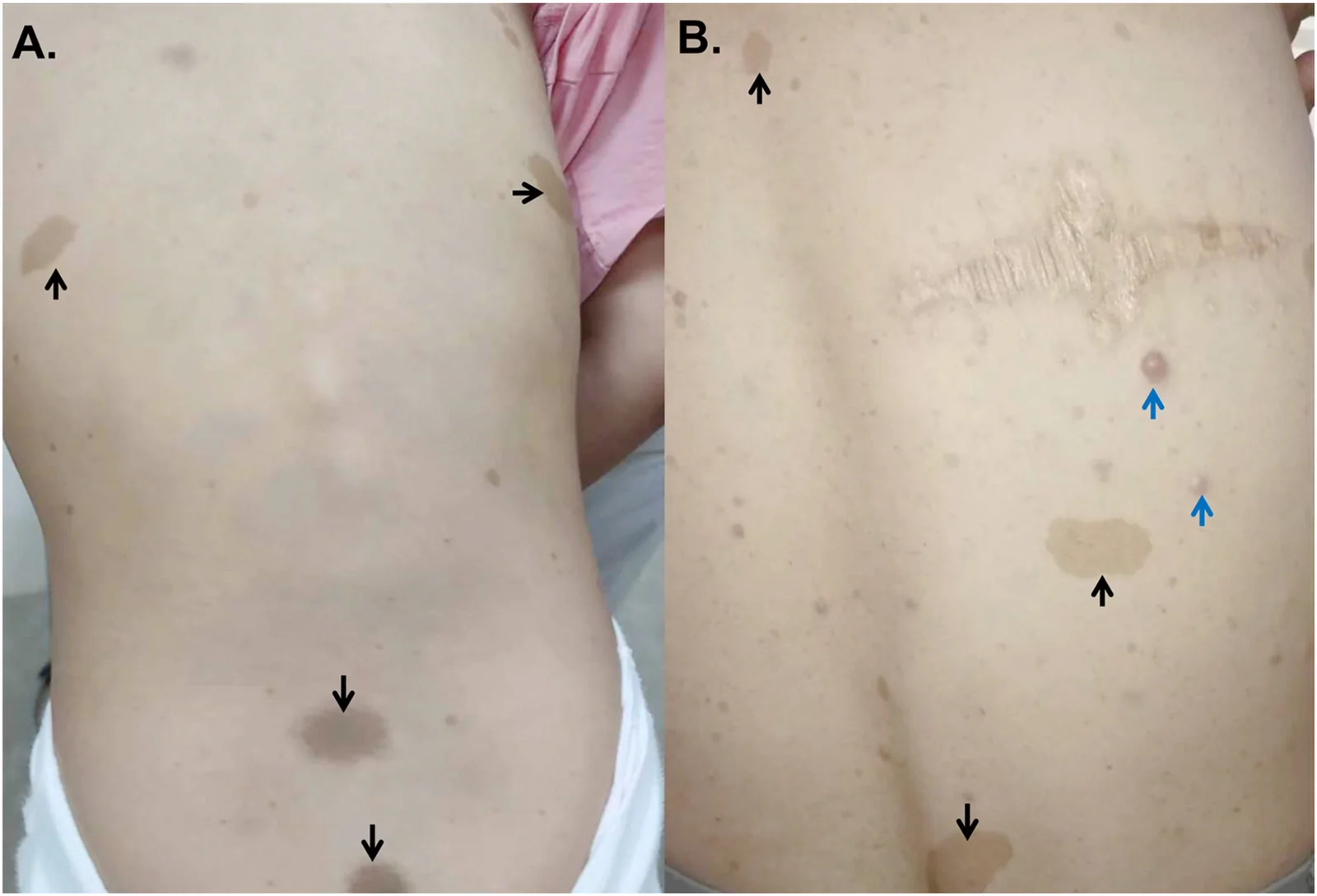
Cutaneous manifestations in the patient and her father. **(A)** Multiple café-au-lait macules on the patient's back (black arrows). **(B)** Café-au-lait spots (black arrows) and neurofibromas (blue arrows) on the father's torso.

Family history was notable: her father exhibited multiple café-au-lait spots (>6) on the trunk, along with numerous soft subcutaneous nodules and a scar from previous neurofibroma resection (Figure 1B). The father denied any history of ocular symptoms, diagnosed glaucoma, or known Lisch nodules, and no formal ophthalmic examination was performed. He exhibited multiple café-au-lait spots and neurofibromas, consistent with a clinical diagnosis of NF1, indicating a positive family history. Genetic testing was not performed in either the patient or her father.

### 2.1 Physical Examination

Multiple café-au-lait macules and axillary freckling were observed on the patient's trunk, with over 10 lesions measuring >5 mm in diameter, which fulfilled the NIH diagnostic criteria for NF1 (7, 11) (Figure 1A).

### 2.2 Ocular Examination:

#### 2.2.1 Right Eye

Best-corrected visual acuity (BCVA) was 20/20 (1.0). IOP was 18.5 mmHg. Slit-lamp biomicroscopy and fundus examination were unremarkable (Figure 3A).

#### 2.2.2 Left Eye

**Figure 2:**
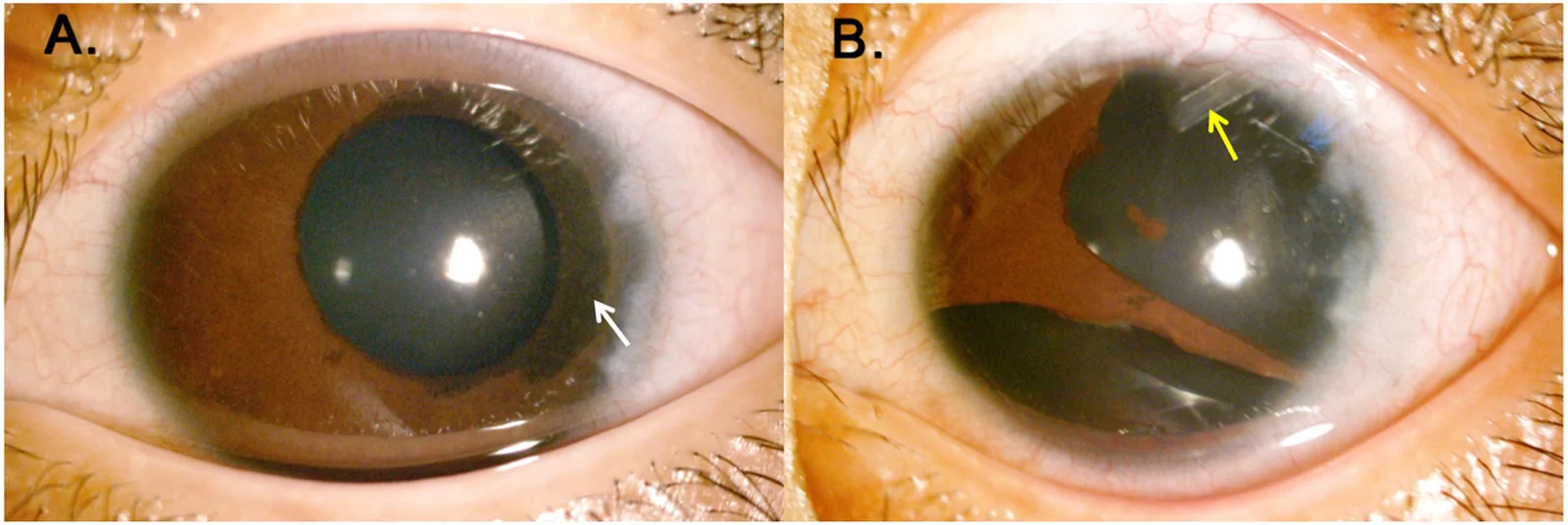
Anterior segment photographs of the left eye. **(A)** A preoperative slit-lamp photograph demonstrating prominent ectropion uveae (white arrow). **(B)** A postoperative view following glaucoma drainage device implantation, with the tube tip visible in the anterior chamber (yellow arrow). The severe iris atrophy and tissue loss observed postoperatively are likely cumulative consequences of prior failed angle surgeries, transscleral cyclophotocoagulation, and chronic ischemic changes due to longstanding elevated IOP, rather than a direct complication of GDD implantation alone.

**Figure 3:**
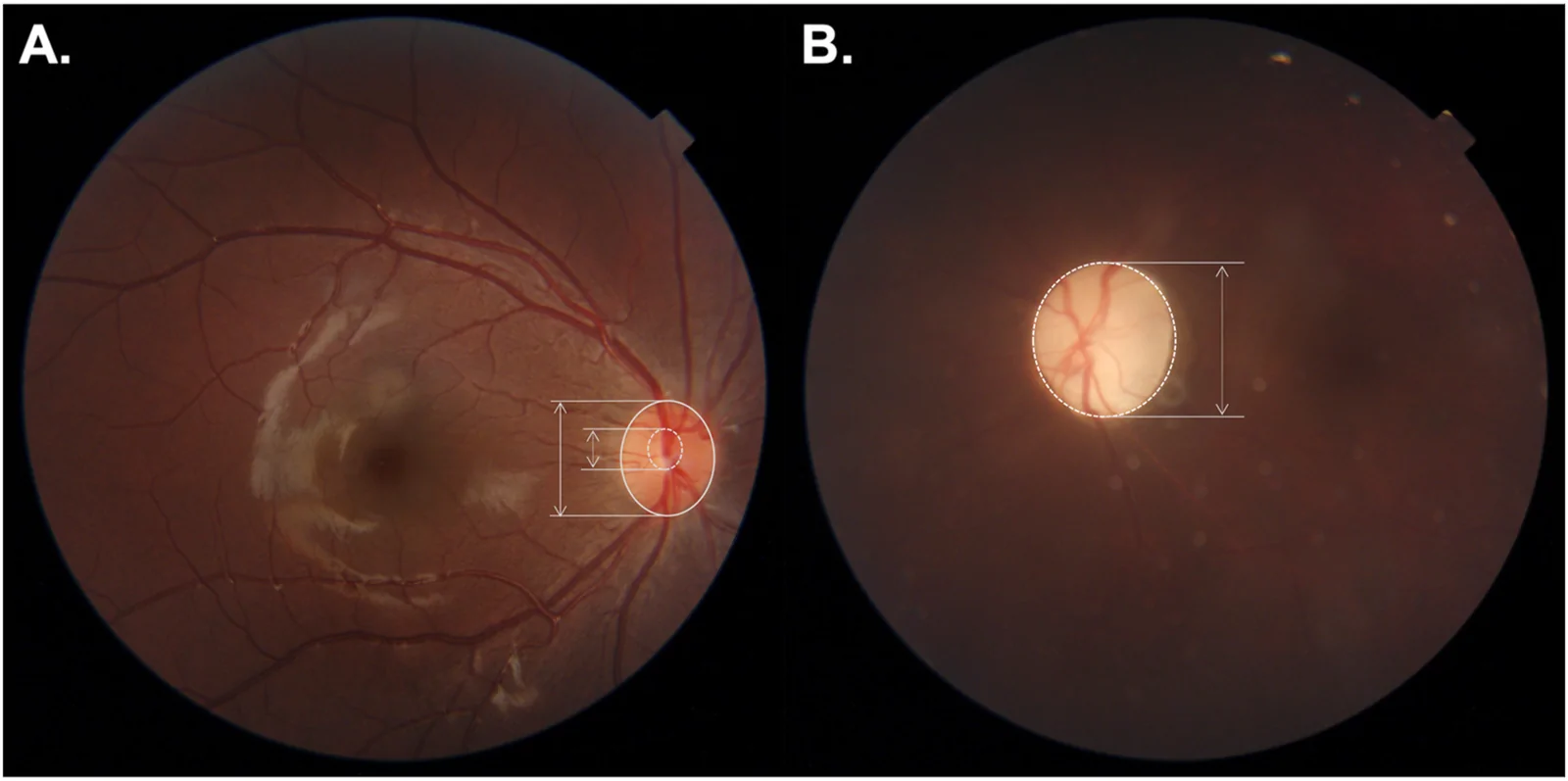
Comparative fundus photography. **(A)** The right eye showing a normal optic disc (C/D ≈ 0.3). **(B)** The left eye revealing end-stage glaucomatous cupping (C/D = 1.0).

BCVA was counting fingers (CF) at 40 cm. IOP was 22.5 mmHg on presentation (under medication). Mild proptosis and 15° of esotropia were noted. The conjunctiva was mildly injected. The cornea was clear but with limbal stretching. The anterior chamber was deep centrally (>6 corneal thicknesses) but shallow peripherally (∼1 corneal thickness). Aqueous flare with pigment granules was present. The iris showed stromal atrophy and marked ectropion uveae (Figure 2A). The pupil was dilated to approximately 6 mm and non-reactive to light. The lens showed mild lens opacity with pigment deposition on the anterior capsule. Funduscopy revealed a pale optic disc with a cup-to-disc ratio (C/D) of approximately 1.0 (Figure 3B). Gonioscopy was not feasible due to poor cooperation (Table 1).

**Table 1:** Baseline ocular characteristics of the patient at initial presentation (June 2021).

Parameter	Right eye	Left eye
Visual acuity	20/20 (1.0)	CF (40 cm)
IOP (mmHg)	18.5	22.5 (on medication)
Optic disc C/D ratio	0.3	1.0
Axial length (mm)	21.81	26.44
Corneal thickness (µm)	576	629
Endothelial cell density (cells/mm^2^)	3,318	2,765
Refractive error	Plano	−6.00 DS/−3.00 DC × 170°

IOP, intraocular pressure; C/D, cup-to-disc.

A multidisciplinary evaluation confirmed the clinical diagnosis of NF1, which was established at the age of 7 years based on the NIH clinical criteria. The ocular diagnoses included secondary glaucoma (left eye), uncontrolled intraocular pressure despite prior surgical interventions, esotropia, amblyopia, and a mild coincident cataract.

## 3 Therapeutic intervention and follow-up

**Figure 4:**
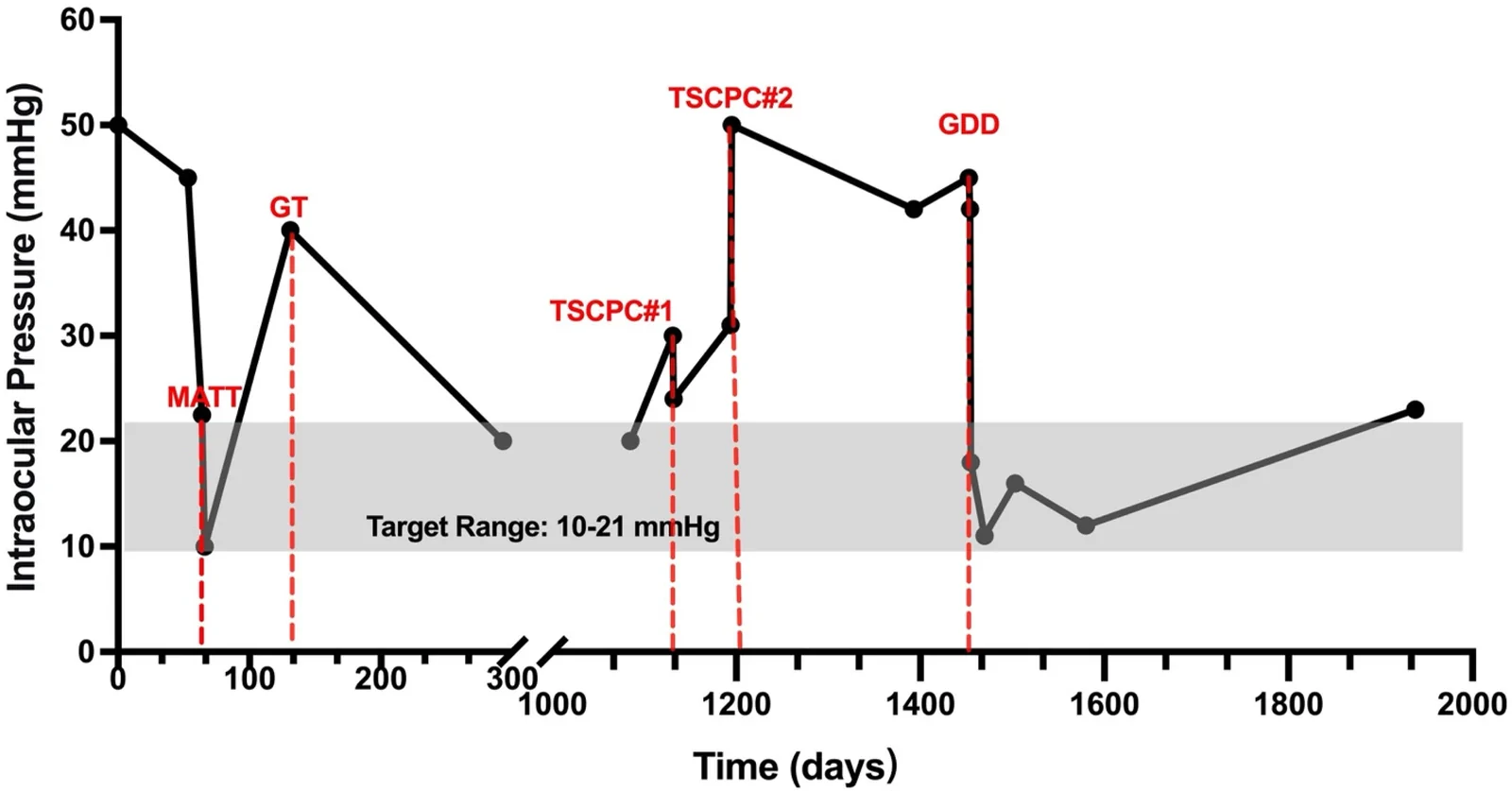
A longitudinal intraocular pressure (IOP) profile and surgical timeline. The graph shows IOP measurements over approximately 1,900 days. Data points are connected by a line. The vertical dashed lines indicate surgical interventions: microcatheter-assisted transluminal trabeculotomy (MATT), goniotomy (GT), first and second transscleral cyclophotocoagulation (TSCPC#1, #2), and glaucoma drainage device (GDD) implantation. The final data point represents IOP at 1-year post-GDD (23 mmHg). The gray shaded area denotes the target IOP range (10–21 mmHg).

The management timeline and corresponding IOP are summarized in Figure 4.

### 3.1 Initial medical therapy

At the referring hospital, the patient had been treated with topical brinzolamide and latanoprost, along with oral acetazolamide and intravenous mannitol, for acute IOP reduction. Despite these measures, IOP remained poorly controlled, prompting surgical intervention.

### 3.2 Initial surgery (July 2021)

Microcatheter-assisted transluminal trabeculotomy (MATT) was performed on the left eye. The procedure was aborted after achieving only 180° of incision because of encountering significant resistance and obstruction in the Schlemm's canal, preventing further catheter advancement. Outcome: Failure to control IOP.

### 3.3 Revision (September 2021)

Excision of fibrotic bleb tissue combined with 60° goniotomy (GT) was conducted. Outcome: Recurrent IOP elevation. Between September 2021 and April 2024, despite continued topical medical therapy (brinzolamide and tafluprost), the patient's IOP ranged between 17 and 23 mmHg, with no further surgical intervention.

### 3.4 Cyclodestruction (April and July 2024)

Because of recurrent IOP spikes above 31 mmHg, two sessions of transscleral cyclophotocoagulation (TSCPC) were performed. However, both failed to provide adequate long-term IOP control.

### 3.5 Definitive management (April 2025)

Despite maximal medical therapy, IOP rose again to 42 mmHg. A glaucoma drainage device (Ahmed FP7 Glaucoma Valve) was implanted in the left eye under general anesthesia. A fornix-based conjunctival flap was created, and mitomycin C (0.4 mg/mL for 4 min) was applied to the scleral bed, followed by thorough irrigation with normal saline. The plate was secured to the sclera between the superior and the lateral rectus muscles. Using a 23-gauge needle, a scleral tunnel was created 4 mm posterior to the limbus at the 1 o'clock position, through which the tube was inserted into the anterior chamber. The tube tip was positioned approximately 2 mm posterior to the limbus. The anterior chamber was stabilized with viscoelastic material, which was subsequently rinsed out. The conjunctiva was closed with interrupted 10-0 nylon sutures (Figure 2B).

### 3.6 Post-GDD course

At 1- and 3-month postoperative visits, IOP remained stable at 16.0 and 18.0 mmHg, respectively, without any glaucoma medications. At the 1-year postoperative visit, BCVA in the left eye had improved to 0.1 (Snellen equivalent 20/200), IOP was 23 mmHg, and brinzolamide was added. The optic disc remained pale with end-stage glaucomatous cupping (C/D = 1.0), and esotropia persisted. The affected eye (OS) exhibited significant progressive axial elongation, from 26.44 mm at initial presentation to 27.13 mm during the 4-year follow-up, despite multiple surgical interventions.

## 4 Discussion

We present a severe, recalcitrant case of secondary glaucoma in a pediatric patient with NF1, distinguished by unilateral buphthalmos, advanced optic neuropathy, and marked EU. The relentless failure of conventional surgical strategies, culminating in IOP reduction only after GDD implantation, offers insights into the pathophysiology and therapeutic challenges of this distinct NF1-associated glaucoma phenotype. At 1-year post-GDD, IOP was 23 mmHg.

**Figure 5:**
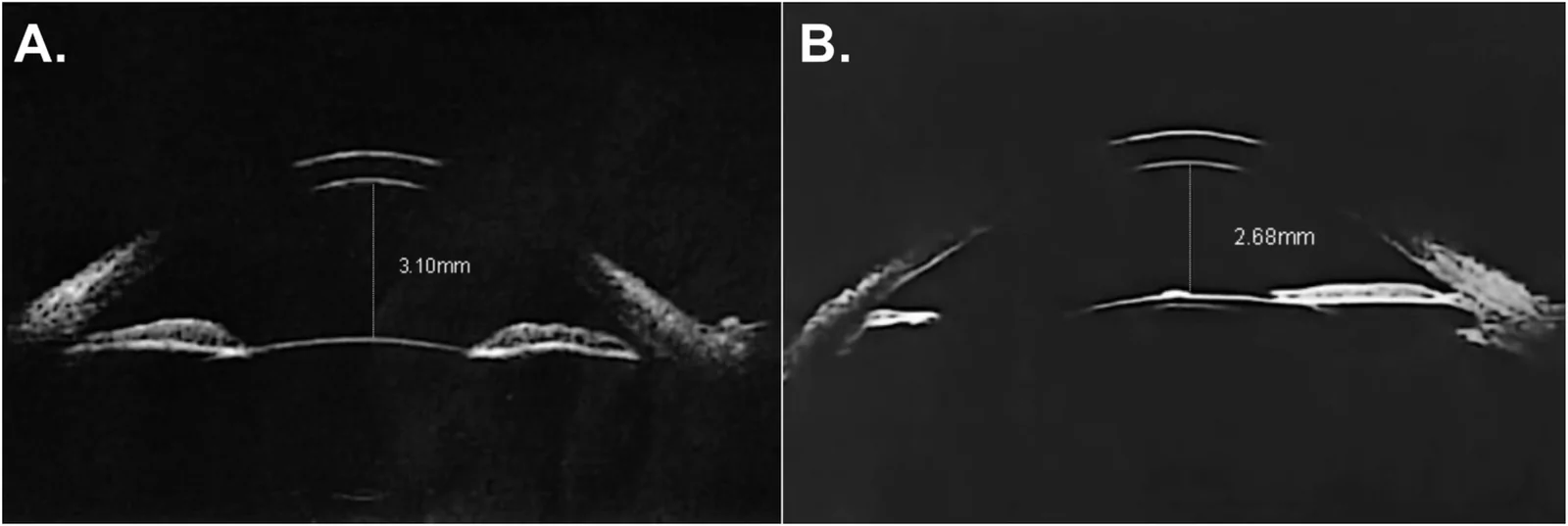
Ultrasound biomicroscopy (UBM) images of both eyes. **(A)** Right eye: Normal open iridocorneal angle and anterior chamber depth (3.10 mm). **(B)** Left eye: A significantly shallow anterior chamber (2.68 mm) with angle closure. A hyperreflective membrane-like structure obscures the angle recess. A UBM image was obtained on 9 April 2025, approximately 4 years after initial presentation.

EU has been reported as a rare but characteristic anterior segment finding in NF1-associated glaucoma, and its presence in our patient appears to be associated with a particularly refractory clinical course (12). Histopathological studies, notably by Edward et al. (6), indicate that EU in NF1 often reflects anterior angle endothelialization. These authors proposed a “contractile membrane theory,” in which a prereceptorial membrane, likely of neural crest origin, extends across the angle and onto the iris. This membrane not only mechanically obstructs the trabecular meshwork but also exerts traction on the pigment epithelium, resulting in the clinical appearance of EU. Crucially, in our patient, anterior segment imaging (Figure 5) provided objective biometric and structural corroboration of this pathological environment. The images revealed a significantly shallower anterior chamber in the affected eye along with hyperreflective alterations at the angle, findings suggestive of profound angle dysgenesis. This mechanism plausibly explains the failure of our initial angle-based procedures, as the pathology entails a physical endothelial barrier and anatomical distortion rather than merely a functional impairment of the meshwork.

Furthermore, the pronounced asymmetry in axial length (26.44 mm vs. 21.81 mm at presentation) aligns with the concept of “globe enlargement” described by Morales et al. (13). Their retrospective analysis identified buphthalmos as a specific marker of NF1-associated glaucoma, noting significantly greater axial length in glaucomatous NF1 eyes compared with nonglaucomatous ones. In our patient, progressive axial elongation (reaching 27.13 mm postoperatively) despite surgical interventions highlights the challenge of managing the highly compliant pediatric sclera, in which elevated IOP directly translates into axial myopia and structural instability, necessitating rigorous IOP-control targets.

Therapeutically, the management of this patient illustrates the notoriously refractory nature of NF1-associated glaucoma complicated by EU. The sequential failure of angle-based and cyclodestructive procedures underscores that in NF1 patients with prominent EU, the standard aqueous outflow pathways are often fundamentally compromised by the underlying angle dysgenesis. Ultimately, implantation of a glaucoma drainage device contributed to IOP reduction in this patient, decreasing from a preoperative peak of 42 to 23 mmHg at 1 year. This outcome is consistent with that of recent case reports where GDD implantation served as the definitive intervention for NF1-related glaucoma with progressive EU and angle closure (10).

Several case reports have described EU in NF1-associated glaucoma, with outcomes ranging from successful GDD implantation (10) to medical management (14) or trabeculectomy (15). Compared with these reports, our case provides a longer follow-up (4 years) and a more detailed record of stepwise surgical failures prior to GDD implantation. These observations raise the possibility that, for similar patients, early implantation of a glaucoma drainage device may be worth considering. However, this hypothesis requires further validation in larger cohorts.

## 5 Study limitations

As a single case report, our conclusions are descriptive. The lack of histopathological confirmation of the angle membrane and comprehensive genetic analysis of the NF1 mutation are limitations. No dedicated cranial or orbital neuroimaging was performed to definitively exclude optic pathway glioma, which represents a clinical limitation of this case report. The postoperative follow-up after GDD implantation was 1 year, which limits the ability to claim long-term durability, particularly as the patient required adjunctive medication after the first year. However, the detailed longitudinal clinical data provide valuable real-world insights into the disease course and management challenges.

## 6 Conclusion

In the case of the patient in this study, prominent ectropion uveae was associated with a refractory surgical course, and conventional angle surgeries repeatedly failed to control IOP. Whether this association applies more broadly requires further investigation. For this patient, GDD implantation contributed to IOP reduction from 42 to 23 mmHg at 1 year, after which adjunctive brinzolamide was required. Longer follow-up is needed to assess long-term durability.

## Data Availability

The raw data supporting the conclusions of this article will be made available by the authors, without undue reservation.
